# Special Issue: Materials, Design and Process Development for Additive Manufacturing

**DOI:** 10.3390/ma15103492

**Published:** 2022-05-12

**Authors:** Vadim Sufiiarov

**Affiliations:** Institute of Mechanical Engineering, Materials, and Transport, Peter the Great St. Petersburg Polytechnic University, Polytechnicheskaya 29, 195251 St. Petersburg, Russia; vadim.spbstu@yandex.ru

Additive manufacturing is a dynamically developed direction of modern digital manufacturing processes, which in some cases is already being used to create high-tech products, and in others there are active investigation on new materials and the design and development of technological processes.

Many additive manufacturing processes are based on the use of highly concentrated energy sources, such as laser, electron beam, microplasma, etc., and imply direct manufacture of parts. The most popular processes use powder materials as raw materials, although wire technologies are also gaining popularity at the moment. Local exposure of high-density energy makes it possible to process materials with a high melting point, such as tungsten [1], and to create complex-shaped products from them that were not previously available for manufacture.

Computer modeling expands the understanding of processes occurring during manufacturing and provides information for further improvement of technology. In the article [2], a model has been proposed for the estimation of parameters of a melt pool in laser metal deposition technology (powder-based directed energy deposition process), and the influence of the residual temperature from the previous deposited layers as well as the temperature of the heated powder in the gas–powder jet, taking into account its spatial distribution are considered. Modeling of the size and shape of the melt pool, as well as the profile of its surface, was performed for various alloys: 316 L stainless steel, Inconel 718 nickel alloy and Ti-6Al-4V titanium alloy.

Heat treatment should be applied for most alloys after additive manufacturing to obtain the desired properties. In the articles [3,4,5], the features of heat treatment on the properties of various application alloys produced by laser metal powder deposition technology are explored. The best mechanical characteristics of additively manufactured Ti-6Al-4V are provided with heat treatment at 900 °C temperatures with a 2-h holding time. The chosen heat treatment mode ensures uniformity of the properties and microstructure in manufactured Ti-6Al-4V samples with variable thickness and complexity, especially in the deposited gas turbine blades [3]. Repeating the cycle of heat treatment twice at T = 620 °C, with a holding time of 2 h for 06Cr15Ni4CuMo steel manufactured by laser powder metal deposition, results in forming a finely dispersed structure of hardened resistant martensite, providing a set of properties equal to the casting material, which makes it promising to use this additive manufacturing process for producing water propellers [4].

The most common technology of powder production for powder-based additive manufacturing processes is gas atomization [6]. At the same time, alternative methods are popular among researchers that allow us to perform synthesis and property evaluation of new materials, with their subsequent application in additive manufacturing processes. The article [7] presents the results of W-C-Co system powder synthesis: In the first stage, agglomerates are formed from nanoparticles by spray-drying technology; then, spherical dense particles are formed by processing in plasma jet. Controlling the plasma treatment process allows us to adjust the technological parameters (average particle size, apparent density, flow rate, etc.) of the synthesized powder material. A similar approach was used by the authors of the article [8] for the synthesis of SiC-SiC system spherical powder for use in the binder jetting additive manufacturing process. The initial non-spherical powders of SiC and Si were used for synthesis, and after spray drying, non-dense agglomerates of 10–80 µm were formed, bonded with a binding agent (PVA). The process of plasma spheroidization led to the melting of Si, which significantly densified particles, and to the decomposition of PVA binder with the formation of carbon. Carbon reacted with silicon to form a secondary SiC. After plasma treatment, the particles had a spherical shape and were 24–90 µm in size. For the binder jetting, the synthesized powder was mixed with SiC fibers. The results of applying the Ti-48Al-2Cr-2Nb intermetallic alloy powders produced by gas atomization and the combination of mechanical alloying and plasma spheroidization technologies are presented in a previous article [9]. The investigation was carried out via selective laser melting technology (laser powder bed fusion process), and the used SLM system made it possible to implement the process with high-temperature preheating of the substrate (600–900 °C). Crack-free samples were fabricated with a 900 °C preheating temperature. Very fine microstructures consisting of lamellar α_2_/γ colonies, equiaxed γ grains, and retained β phase were obtained in all samples. Aluminum loss during the selective laser melting process led to a shift in the solidification route and resulted in the formation of the retained β phase. Increased oxygen content in the initial powder led to the formation of small oxides and an increased α_2_ volume fraction. The samples fabricated from gas atomized powder demonstrated superior compressive performance compared to the samples from the mechanically alloyed plasma spheroidized powder. Both alloys showed superior compressive properties compared to the conventional TiAl-alloy. The in situ synthesis approach is often used in additive manufacturing research as an economical method for evaluating microalloying on known compositions, and [10] presented a study of adding niobium to nitinol powder. Despite the incomplete melting of niobium particles, it was possible to achieve the formation of a dense material, with subsequent heat treatment at 900 °C with 2 h of dwell allowing increasing martensitic transformation hysteresis as compared to the alloy without Nb addition from 22 to 50 °C, while the A_f_ temperature increased from −5 to 22 °C.

A trend that is attracting more and more research attention is multimaterial printing with metals, shown in [11], where different geometries of interfaces, the effects of heat treatment and hot isostatic pressing on the microstructure of intermaterial zones, and mechanical properties were studied. The researchers found that when intermaterial zones are located along the sample, the ratio of the cross-section has greater influence on the mechanical properties than their shape and location. When the zones are arranged transversely to the specimen, a failure occurs at the interface and relative elongation is extremely low.

Some additive manufacturing processes include indirect fabrication, and the formation of the final properties during that type of manufacturing requires applying special post-processing methods (sintering, infiltration, reaction sintering, etc.). Such approaches are used for the additive manufacturing of ceramic materials, as well as for metals [12,13] and composites [14]. The article [8] presents the results of an investigation of manufacturing SiC_f_-SiC ceramic composite materials. For the manufacture, the binder jetting process was used, after which densification by infiltration and pyrolysis were performed. Polycarbosilane preceramic polymer was used for vacuum infiltration, which has good wettability and small pore filling ability, and after infiltration, the samples were heated up to 1000 °C with dwell for 1 h. As a result, polycarbosilane pyrolysis occurred with the formation of silicon carbide and densification of the samples. The infiltration and pyrolysis procedures were carried out several times. The greatest densification was observed after the first few cycles, and then the rate of rising density (decreasing porosity) became lower. In the article [15], the binder jetting process was used for the fabrication of ceramic green models and pressureless sintering for densification. The effect of sintering modes on the relative density and grain sizes of samples made of two types of materials (submicron powder with unimodal particle-size distribution and micron powder with multimodal particle-size distribution) is presented.

Another promising area is the investigation of materials and processes for the additive manufacturing of functional materials. Shape memory materials are now being actively researched for production by additive manufacturing and expanding possibilities of their application by corrections of alloying systems [10]. Particularly relevant in the global trend towards green energy are materials used in electrical machine component production, such as electricity conductors, soft-magnetic alloys, piezoceramics, etc. Additive manufacturing with such materials is a very current and attractive direction of investigation. Research into the structure and properties of lead-free piezoelectric ceramics based on barium titanate produced by the binder jetting process is presented in the article [15], and the results demonstrate that the use of barium titanate allows achieving high piezoelectric properties, and using binder jetting allows the creation of objects with complex geometry, which has great potential in the manufacture of ultrasonic products used in medicine, aviation, the marine industry, sensors for monitoring welded joints, pressure sensors in pipelines, etc. In [16], three strategies for improving the magnetic properties of Fe-based soft magnetic materials manufactured by selective laser melting have been suggested. The first one is to optimize the parameters of the selective laser melting process for alloys whose chemical composition is chosen to achieve the maximum values of electrical resistivity. Using optimized process parameters and heating of the SLM substrate, the authors obtained a crystalline sample of FeSi6.7 alloy with the minimum coercivity of H_c_ = 16 A/m and hysteresis losses of 0.7 W/kg at 1 T and 50 Hz. The second one is to optimize the geometry of samples by designing different inner hollow structures into samples. This allows us to reduce eddy current losses in a magnetic field by changing the path of the electric current. The third strategy is to form a sample consisting of alternating layers of soft-magnetic material separated by electrically insulating material (multimaterial printing). The strategies may be combined, which allows more flexible manage properties. Active investigations into the additive manufacturing of amorphous and nanocrystalline soft-magnetic alloys is under development at this time. This type of material has a significantly higher level of soft-magnetic properties compared to crystalline analogues. High cooling rates are typical for the selective laser melting process, but to reduce crystallinity and increase magnetic properties sometimes it is necessary to use nonstandard build strategies of laser scanning [17]. The first strategy was double scanning of each layer, the use of which enabled the researchers to achieve a maximum relative density of 96% and saturation magnetization of 1.22 T. The usage of this scanning strategy also increased the amorphous phase content of samples to 47% and reduced coercivity. The second strategy consists of a two-stage powder melting process: pre-laser melting and short-pulse laser treatment for amorphization. The application of this strategy resulted in a sample containing 89.6% amorphous phase and having a relative density of up to 94.1%.
